# A case report of a patient with advanced gastric adenocarcinoma who demonstrated excellent long-term sustained efficacy even after discontinuation of treatment following chemotherapy combined with claudin18.2 and PD-1 therapy

**DOI:** 10.3389/fphar.2026.1612299

**Published:** 2026-02-25

**Authors:** Siyu Yu, Hong Zhu

**Affiliations:** Department of Medical Oncology, Cancer Center, West China Hospital, Sichuan University, Chengdu, Sichuan, China

**Keywords:** gastric cancer, claudin 18.2, epstein-barr virus (EBV), discontinuation of treatment, excellent efficacy

## Abstract

Gastric cancer is the fifth most common cancer and the third most common cause of cancer death globally. Patients with advanced gastric cancer have poor outcomes and short survival times. This case report presents a remarkable clinical response in a patient with advanced Epstein-Barr virus (EBV)-associated gastric adenocarcinoma treated with a combination of chemotherapy, Claudin18.2-targeted therapy (TST001), and PD-1 inhibition (nivolumab). The patient, initially diagnosed with stage IA disease (pT1bN0M0) after Billroth II gastric resection was performed due to early carcinoma, later developed metastases to the liver, cervicothoracic lymph nodes and abdominal lymph nodes. The patient received seven cycles of CAPOX (capecitabine + oxaliplatin), nivolumab, and TST001, achieving partial response (PR) after treatment. Treatment was discontinued due to aortic dissection requiring surgery. Surprisingly, despite no further antitumor therapy, follow-up imaging over 19 months revealed continued tumor shrinkage, culminating in a near complete response (CR). Therefore, the combination of chemotherapy, Claudin18.2-targeted therapy, and PD-1 inhibitor may be a good treatment strategy for gastric cancer.

## 1 Introduction

Gastric cancer is the fifth most common malignancy worldwide, with particularly high incidence rates in East Asian countries. China has consistently ranked among the highest in both global incidence and mortality rates of gastric cancer, which may be attributed to the low rate of early screening in the country (Joshi and Badgwell, 2021). Many patients are often diagnosed at advanced stages, resulting in extremely poor prognosis. For early-stage gastric cancer patients, the primary treatment relies on perioperative chemotherapy and surgery. However, gastric cancer is highly aggressive, with a very low survival rate—typically less than 1 year for advanced cases. In the past, the ToGA trial established trastuzumab plus chemotherapy as the standard first-line treatment for advanced HER2-positive gastric adenocarcinoma (Bang et al., 2010). Compared to chemotherapy alone, the addition of trastuzumab improved median overall survival (OS). However, due to the low HER2 positive rate, the population benefiting from this regimen was limited, and subsequent attempts with various targeted therapies proved disappointing.

In recent years, The emergence of immune checkpoint inhibitors has greatly improved the prognosis of patients with advanced gastric cancer, and the patient efficacy and survival time have been significantly improved (Tarantino et al., 2022), leading to improved survival rates in gastric cancer patients. Despite these advances, the overall survival rate for gastric cancer remains poor, underscoring the urgent need for more effective treatment strategies. Claudin 18.2 is a protein highly expressed in gastric cancer, panrcreatic cancer and so on. Studies have shown that claudin 18.2-targeted therapies demonstrate significant clinical benefits in claudin 18.2-positive and HER2-negative gastric cancer patients in both the SPOTLIGHT and GLOW trials. However, they only evaluated the efficacy of adding a single claudin 18.2-targeted drug in gastric cancer treatment (Shah et al., 2023; Shitara et al., 2023). Currently, there is still a lack of large-scale Phase III trials demonstrating the benefits of combining of chemotherapy, Claudin18.2-targeted therapy, and PD-1 inhibitor immunotherapies in gastric cancer treatment. Here, we present a case of a patient who underwent a chemotherapy + immunotherapy + Claudin18.2 targeted therapy regimen. After seven treatment cycles, a follow-up CT scan revealed an aortic dissection, prompting treatment discontinuation for surgical intervention. After aortic surgery, the patient ceased all antitumor therapy. Remarkably, even after 19 months without any cancer treatment, the tumor continued to shrink, eventually achieving a near complete response (CR).

## 2 Case presentation

**FIGURE 1:**
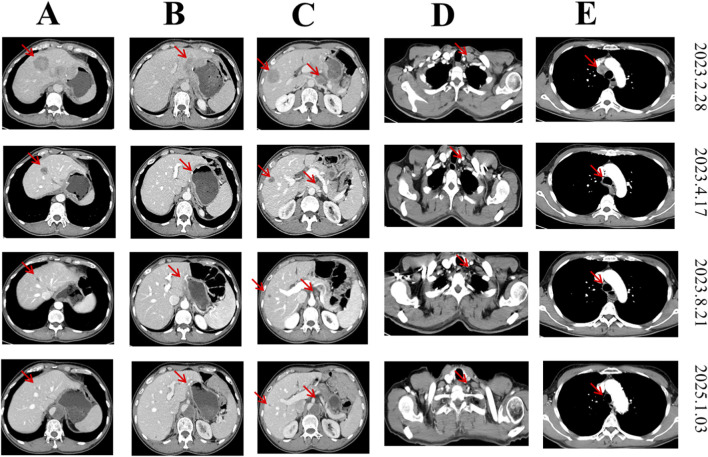
Representative tumor images before and after treatment. The red arrow points to the tumor. 18 March 2023 imaging (prior to initiation of CAPOX combined with nivolumab and TST001 treatment) revealed baseline lesion status. 17 April 2023 imaging (after 2 cycles of the treatments) showed partial response (PR). 21 August 2023 imaging (after 6 cycles of treatments) demonstrated continued tumor shrinkage. 3 January 2025 imaging (19 months after discontinuation of the last treatment) revealed long-term follow-up findings. **(A)** The changes of liver metastatic lesions before and after treatment; **(B)** Comparison of residual gastric cancer lesions before and after treatment; **(C)** Comparison of another liver metastatic lesion with concurrent abdominal lymph node metastasis; **(D)** The changes in cervical lymph node metastatic lesion; **(E)** Comparison of mediastinal lymph node metastasis before and after treatment.

In January 2019, a male patient of 57 years old was diagnosed with gastric cancer due to mid-upper abdominal pain with acid reflux and belching at the time of treatment in our hospital. The patient is 160 cm in height and weighs 50.5 kg. And the patient was in a generally good condition. Physical examination of the head, neck, chest, and abdomen revealed normal contours. An old scar was noted on the abdominal wall. The abdomen was soft overall, without tenderness or rebound tenderness. Bowel and bladder functions were normal, though the patient reported poor sleep quality. Since the onset of the illness, the patient has experienced symptoms of acid reflux, belching, and loss of appetite. Then the patient underwent “radical distal Billroth II gastric resection with D2 lymphadenectomy” in our hospital, during which it was found that the abdominal tumor was located on the side of the greater curvature of the gastric antrum, about 2*2 cm, ulcerative, not penetrating the serous layer, and several enlarged lymph nodes could be reached in the antrum, hepatoduodenal ligament, and hypogastric ligament. Postoperative pathology: early cancer: type III. Tumor size: 2 cm × 1.3 cm × 0.4 cm. Microscopic tumor invasion depth: invasion of the submucosa. Histology type: Special type: EBV-associated adenocarcinoma (lymphepithelioma-like carcinoma). Immunohistochemical staining results: HER2 (0), PD-1 (lymphocyte individual), histological grade: medium ∼ low differentiation. The results of immunohistochemical staining for the detection of mismatch repair proteins were MLH-1 (+), PMS2 (+), MSH2 (+), and MSH6 (+). Subsequently, the following therapy-related targets were detected: CPS of PD-L1 staining was 3; and the expression of claudin18.2 in this patient was negative (claudin18.2 1+ 10%). The tumor stage is pT1N0M0. The patient did not receive any treatment after surgery and regular CT examinations were performed. Tumor recurrence, suspected on 2 February 2023, during a routine examination at an external hospital, was confirmed by a CT scan performed at our institution on 28 February 2023 (Figure 1 first row). And the gastroscopy showed that the tumor was found in the residual stomach, and the pathology report showed that the tumor was found in the residual stomach, combined with HE morphology and immunophenotype, it supported poorly differentiated adenocarcinoma. Based on the above information, the patient was finally diagnosed with recurrence of the residual stomach with metastases to liver, cervicothoracic lymph nodes and abdominal lymph nodes (Figure 1, first row). On 14 February 2023, the patient voluntarily participated in a “Phase I clinical study to evaluate the safety, tolerability, pharmacokinetics, and preliminary efficacy of Claudin18.2 monoclonal antibody-TST001 in the treatment of locally advanced unresectable or metastatic solid tumors” and started the first treatment on 7 March 2023. After two cycles of treatment, the CT scan on 17 April 2023 showed that all the tumor lesions were significantly reduced (Figure 1, second row). And the treatment efficacy was assessed as a partial response (PR) according to the Response Evaluation Criteria for Solid Tumors (RECIST) 1.1 criteria.

**FIGURE 2:**
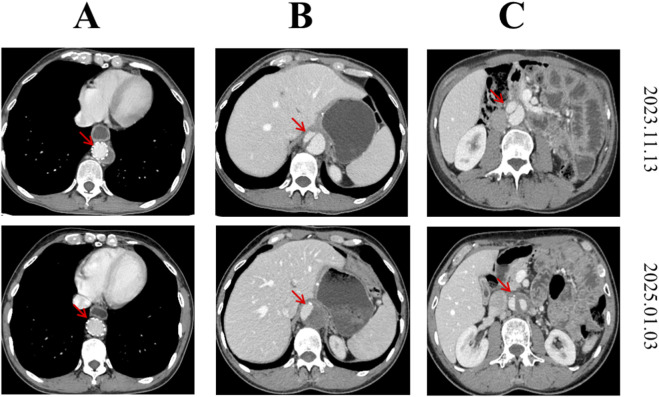
Typical images of aortic dissection 2 months after aortic stent surgery (2023-11-13) and 15 months after aortic stent surgery (2025-1-3). **(A)** Aortic dissection stent, **(B)** Abdominal aortic dissection of liver level, **(C)** Abdominal aortic dissection of kidney level. The figure indicated there were still aortic dissection after surgery and became more serious.

**FIGURE 3:**
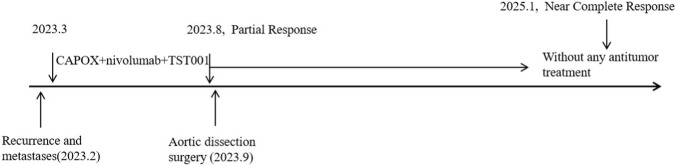
The schematic treatment timeline of the patient. CAPOX: oxaliplatin and capecitabine.

After 6 cycles, the patient’s follow-up CT results (21 August 2023) showed that the tumors continued to shrink (Figure 1, third row). However, after 7 cycles of treatment, the patient discovered an aortic dissection on 4 September 2023 because of severe chest and abdominal pain in another hospital, and aortic stent implantation surgery was performed on 12 September, 2023. After surgery, the patient withdrew from the clinical study. Until now, he did not receive any anti-tumor therapy because the aortic dissection still exist and became worse 2 months after aortic stent surgery and 15 months after aortic stent surgery (Figure 2). What’s surprising is that the patient in January 2025, routine CT examination 19 months after the last treatment showed the residual stomach recurrence, cervicothoracic lymph nodes metastases and abdominal lymph nodes metastases completely disappeared. Most of the liver metastases disappeared. There were only two very small liver metastases left (about 0.3 cm in diameter). So, the efficacy evaluation is close to CR after 19 months without any antitumor treatments. The high recurrence and metastasis rates of gastric cancer have always been a problem in the medical community, but in this patient, he has stopped treatment for such a long time, the tumor has continued to shrink, and the investigator assessed that the target tumor has near CR. At present, the patient has survived for 31 months after treatment with this regimen. The schematic treatment timeline of the patient was showed in Figure 3.

Throughout the duration of tumor treatment, the patient experienced one degree of leukocyte decline, one degree of anemia, third degree of leukocyte decline, and one degree of elevated aminotransferases grade 3 neutrophil degeneration, mild numbness of hands and feet throughout the treatment. There is slight nausea and vomiting.

## 3 Discussion

Gastric cancer ranks as the fifth most common malignancy globally and the third leading cause of cancer-related deaths. The first-line treatment for advanced metastatic gastric cancer primarily consists of platinum-fluoropyrimidine doublet therapy or triplet combination chemotherapy (FLOT regimen) (Joshi and Badgwell, 2021). Despite advances in immunotherapy and targeted therapy, many targeted therapies for gastric cancer still have unsatisfactory effects. The ToGA trial has confirmed for the first time the significant efficacy of trastuzumab in HER2 positive advanced gastric cancer, thereby establishing the status of trastuzumab combined with chemotherapy as the standard first-line treatment for this patient population (Bang et al., 2010). This study has advanced the development of precision medicine in cancer and highlighted the critical importance of HER2 testing in clinical management. However, treatment outcomes remain limited for advanced cancer patients without actionable targets. The emergence of claudin 18.2 has provided a novel therapeutic option for a specific patient subset (Claudin 18.2-positive/HER2-negative). The high expression and tumor-specificity of claudin 18.2 make it an ideal target for precision cancer therapy (Qi et al., 2022).

Claudin18.2 (CLDN18.2), a subtype of the tight junction protein family member claudin18, is a highly selective biomarker. It plays a crucial role in tumor cell proliferation, differentiation, and migration (Cao et al., 2022). The protein features two extracellular loops that bind to CLDN18.2 molecules expressed on adjacent cell surfaces, forming selective permeability barriers that maintain tissue-specific permeability and support the polarity of gastric epithelial cells (Cao et al., 2022). Due to its unique expression pattern, CLDN18.2 has emerged as a distinctive molecular target for precision therapy across various cancers. The clinical SPOTLIGHT and GLOW trials indicated that the combination of Zolbetuximab (a CLDN18.2 inhibitor) and chemotherapy could significantly improve the prognosis in patients with CLDN18.2-positive, HER2-negative, locally advanced unresectable or metastatic gastric or gastro-oesophageal junction adenocarcinoma. In SPOTLIGHT trial, Zolbetuximab treatment showed a significant reduction in the risk of disease progression or death compared with placebo. The median progression-free survival was 10.61 months in the zolbetuximab group versus 8.67 months in the placebo group. Zolbetuximab treatment also showed a significant reduction in the risk of death versus placebo. In GLOW trial, Zolbetuximab treatment also significantly improved the primary endpoint of progression-free survival (median, 8.21 months versus 6.80 months with zolbetuximab versus placebo; hazard ratio (HR) = 0.687; 95% confidence interval (CI), 0.544–0.866; P = 0.0007) and key secondary endpoint of overall survival (median, 14.39 months versus 12.16 months; HR = 0.771; 95% CI, 0.615–0.965; P = 0.0118).

However, both SPOTLIGHT and GLOW trials did not add PD-1 or PDL-1 inhibitor in the treatment. Besides, all the patients included in these two trials are claudin 18.2 positive (CLDN18.2-positive was defined as ≥75% of tumor cells with moderate-to-strong membranous CLDN18 staining as determined by central immunohistochemistry). In our report, the expression of claudin18.2 in this patient was negative (claudin18.2, 1+, 10%). So our report is the first reported case of combined treatment of chemotherapy, claudin18.2-targeted therapy and PD-1 inhibition, especially in gastric cancer patient with claudin 18.2 low expression.

Epstein-Barr virus (EBV) positivity is a molecular subset of gastric cancer with a unique molecular signature (Yang et al., 2020). In a prospective phase 2 clinical trial, all patients with EBV-positive GC treated with pembrolizumab achieved a partial response (PR), with a longer median duration of response of 8.5 months. This study suggests that the combination of EBV and PD-L1 may be a more accurate combination of biomarkers to determine the efficacy of immunotherapy on GC (Kim et al., 2018). However, a subsequent single-arm phase 2 prospective clinical trial enrolled 6 patients with EBV-positive mGC treated with camrelizumab, but none achieved an objective response (Hou et al., 2023), raising doubts about EBV-positivity as a reliable predictor of mGC immunotherapy response, and whether EBV-positive is a factor in improving survival in advanced gastric cancer remains to be considered. In the Asian ATTRACTION-2 study, PD-L1 (+), low NLR and normal Na (≥135 mmol/L) were associated with higher response and disease control rates in the nivolumab arm, while tumor EBV infection and TMB were not correlated (Kim et al., 2022). There is also a lack of conclusive clinical studies to prove the beneficial effect of anti-PD-1 treatment in EBV positive gastric cancer. In this case, the patient is a EBV positive gastric cancer patient. It maybe one part of good efficacy. However, the sustained treatment efficacy after 19 months of treatment discontinuation is also incredible.

In this case, the patient was treated with Osemitamab (TST001), a high-affinity humanized monoclonal antibody targeting CLDN 18.2 (Katoh and Katoh, 2024). After 7 cycles of TST001 treatment, the treatment efficacy achieved PR according to the Response Evaluation Criteria for Solid Tumors (RECIST) 1.1 and analyzed the results of enhanced CT scan, and even after 19 months of treatment discontinuation, the tumor lesions continue to shrink, which is incredible in patients with advanced gastric cancer. The treatment efficacy is close to CR now. Osemitamab (TST001) may play an important role in the treatment. However, the expression of claudin 18.2 was low in this patient. So, maybe there are synergistic effects of claudin 18.2 target therapy and PD-1 inhibitor treatment. Until now, there is no clinial trial reported combined effects of claudin 18.2 target therapy and PD-1 inhibitor treatment. But in a preclinical study, Nishibata et al. (2024) found that mice treated with zolbetuximab (a CLDN18.2 inhibitor) plus chemotherapy displayed a significantly higher frequency of tumor-infiltrating CD8+T cells versus vehicle/isotype control-treated mice. Furthermore, zolbetuximab combined with an anti-mouse programmed cell death-1 antibody more potently inhibited tumor growth compared with either agent alone. These results showed claudin 18.2 target therapy may enhance the antitumor effects of PD-1 immunotherapy. The case reported here is an EBV- positive gastric cancer patients. Although conclusions about EBV as a novel biomarker for immunotherapy were limited and need more validation, 9% of patients with GC have EBVaGC, and 80% of them harbour an immune-inflamed microenvironment with enriched T-cell and B-cell infiltration (Chong et al., 2024). This may also explain the reason that the patient in this report showed good efficacy after combined treatments of chemotherapy, claudin 18.2 target therapy, and PD-1 inhibitor treatment (Matsuishi et al., 2024). These mechanisms, along with potential synergistic effects of multimodal treatment, may explain the clinical benefit observed in this patient.

## 4 Conclusion

The combined treatments of chemotherapy, claudin 18.2 target therapy, and PD-1 inhibitor treatment showed excellent efficacy in this EBV positive gastric cancer patient even with low expression of claudin 18.2. The patient stopped all the antitumor treatment for 19 months because of aortic dissection, however, all the cancer lesions continued to shrink, and the treatment efficacy is close to CR now. The combination of chemotherapy, anti-Claudin18.2 and anti-PD-1 treatment maybe an effective first line treatment for gastric cancer.

## Data Availability

The original contributions presented in the study are included in the article/supplementary material, further inquiries can be directed to the corresponding author.
